# Systemic Capillary Leak Syndrome as an Initial Presentation of ALK-Negative Anaplastic Large Cell Lymphoma

**DOI:** 10.1155/2012/954201

**Published:** 2012-03-26

**Authors:** Laura S. Lourdes, Samer Z. Al-Quran, Nam H. Dang, Merry-Jennifer Markham

**Affiliations:** ^1^Department of Medicine, University of Florida College of Medicine, Gainesville, FL 32610-0278, USA; ^2^Department of Pathology, Immunology, and Laboratory Medicine, University of Florida College of Medicine, Gainesville, FL 32610-0278, USA; ^3^Division of Hematology and Oncology, Department of Medicine, University of Florida College of Medicine, P.O. Box 100278, Gainesville, FL 32610-0278, USA

## Abstract

Systemic capillary leak syndrome (SCLS) is a rare disease characterized by third spacing of plasma into the extravascular compartment, leading to anasarca, hemoconcentration, and hypovolemic shock. It has been rarely associated with lymphomas, and reports usually indicate that it occurs after antineoplastic treatment. We present the case of a patient with ALK-negative anaplastic large cell lymphoma who presented with SCLS as the initial manifestation of her lymphoma. The SCLS resolved with treatment of the malignancy with steroids and chemotherapy.

## 1. Introduction

Systemic capillary leak syndrome (SCLS) is a rare disease, first described in 1960 by Clarkson et al. [1] It is characterized by third spacing plasma into the extravascular compartment, leading to intravascular volume depletion and giving rise to anasarca, hemoconcentration, and hypovolemic shock. Although about 126 cases are described in the literature, less than ten are lymphomas with SCLS as the presenting feature [2]. We report a case of a patient with ALCL who presents with SCLS that subsequently resolves with steroid therapy followed by chemotherapy.

## 2. Case Presentation

A 71-year-old African American woman presented with a one-week history of lower extremity and facial swelling, nausea, vomiting, and diarrhea. On admission, she was noted to have hypotension (systolic blood pressure 70 mmHg), acute renal failure (blood urea nitrogen 51 mg/dL and serum creatinine 4.99 mg/dL), and hypoalbuminemia (albumin 3 mg/dL). She required fluid and vasopressor resuscitation. Empiric treatment with broad-spectrum antibiotics was started. By hospital day 14, she developed anasarca, 60 pound weight gain, and required additional vasopressor support and continuous veno-venous hemodialysis (CVVH). Evaluations revealed negative or normal blood and urine cultures, quantitative immunoglobulins; serum protein electrophoresis and immunofixation studies, antinuclear antibody, antineutrophil cytoplasmic antibody, anti-double-stranded DNA levels; erythrocyte sedimentation rate. Urine sodium was less than 25 mmol/L, and urine creatinine was 205 mg/dL. She demonstrated adequate response to cosyntropin. Transthoracic echocardiography and right heart catheterization revealed normal left ventricular ejection fraction, normal pulmonary artery wedge pressure, and no pulmonary hypertension. Computed tomography (CT) revealed left inguinal and retroperitoneal lymphadenopathy (largest node measuring 2.2 cm). Positron emission tomography showed hypermetabolic activity of left pelvic lymph nodes.

**Figure 1:**
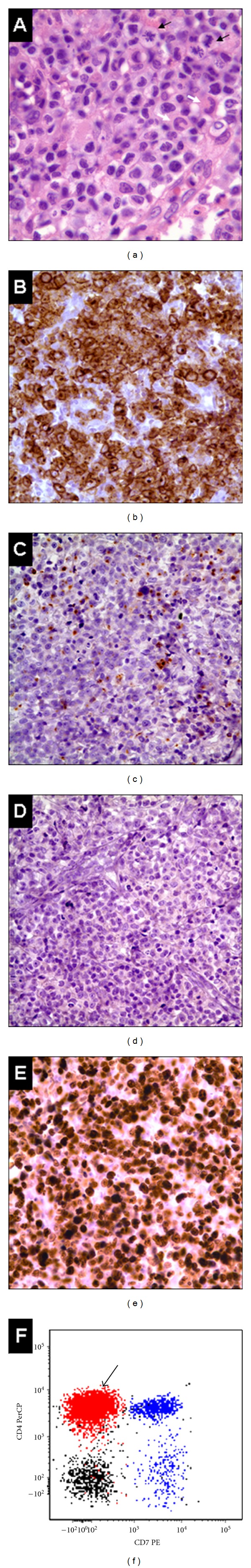
Characteristic features of this case of anaplastic lymphoma kinase-negative (ALK-) anaplastic large cell lymphoma (ALCL). (a) Large pleomorphic neoplastic cells with abundant cytoplasm, eccentric nuclei, and brisk mitotic activity (black arrows); Hallmark cells with horseshoe- or kidney-shaped nuclei are present (white arrows). (b) Neoplastic cells show strong membrane and Golgi expression of CD30. (c) Focal immunoreactivity with antibody for the cytotoxic granule protein, Perforin. (d) ALK expression is lacking in this case. (e) High Ki-67 expression, denoting a worse prognosis. (f) Correlated multiparametric flow cytometric analysis demonstrating an abnormal population of T cells (in red) with expression of surface CD4 and lacking surface CD7. Normal T cells (in blue) show normal expression of CD7 and CD4 and include a CD4− subset, which corresponds to the CD8+ T cells. (Black: B cells).

 Histopathology from core-needle biopsy of the left inguinal lymphadenopathy showed an abnormal infiltrate of relatively cohesive clusters of large pleomorphic cells with morphologic features summarized in Figure 1. Limited flow cytometric analysis was performed and demonstrated the presence of an abnormal population of large T cells with slightly dimmed expression of surface CD3 and CD2 in comparison to the normal small T lymphocytes. These cells showed bright expression of CD45, CD4, CD5 with no expression of CD7, CD8, CD19, CD20, CD10, kappa or lambda surface light chain. DNA analysis by DRAQ5 showed a relatively high tumor-specific S-phase fraction (12.7%).

Immunohistochemical studies showed strong membranous and perinuclear “Golgi” expression of CD30 in nearly all neoplastic cells, which were also ALK (−), CD4 (+), CD8 (−), UCHL1 (+), CD43 (+), EMA (−), TdT (−), cytokeratin AE1/AE3 (−), and S-100 (−). There was frequent labeling of nuclei by Ki-67 (in some areas >90%). The overall findings were consistent with ALK-negative anaplastic large cell lymphoma (ALK-ALCL). Bone marrow evaluation was not performed due to rapidly declining performance status.

She was treated with methylprednisolone 90 mg intravenous twice-daily beginning on day 17. Over the next 10 days, she had spontaneous diuresis, resolution of hypotension, and normalization of renal function, and CVVH was discontinued. On hospital day 24, she received cycle one of cyclophosphamide, vincristine, doxorubicin, and prednisone (CHOP). She was discharged home on day 29.

The patient competed six cycles of CHOP chemotherapy and attained a complete response by CT imaging. Five weeks after completion of chemotherapy, she developed blurry vision. She was found to have central nervous system (CNS) relapse of ALCL with involvement of the cerebrospinal fluid. Despite intrathecal and intravenous methotrexate therapy for one month, she had progressive disease and died two months after CNS relapse. SCLS never recurred after her initial presentation. 

## 3. Discussion

Since its recognition half a century ago, the underlying pathophysiology of SCLS remains uncertain. All patients have similar clinical presentation, but laboratory data, underlying etiology (or lack of), and outcomes have been inconsistent. SCLS has been reported after infusions of interleukin-2 (IL-2) and tumor necrosis factor and has been described in association with non-Hodgkin lymphomas (NHL), multiple myeloma, and following G-CSF mobilization of peripheral blood progenitor cells [3]. 

The most common lab abnormality in idiopathic SCLS is monoclonal IgG paraproteinemia in approximately 80% of patients [2]. Although the level of paraprotein varies by patient, it varies little within the same patient during attacks and remissions [4].

 The occurrence of IgG paraproteinemia is increased in patients with SCLS compared to normal controls, suggesting that it may be implicated in the pathogenesis of SCLS [5].The incidence of SCLS has increased with the advent of interleukin-2 (IL-2) therapy for malignant melanomas and renal cell carcinomas, and with denileukin diftitox (fusion protein with diphtheria toxin and IL-2) for cutaneous T-cell lymphoma. SCLS is the most serious adverse effect of moderate-to-high doses of IL-2, and it begins to reverse within 24 hours of discontinuation of IL-2 therapy and completely resolves within a few days [6]. 

IL-2-induced vascular leak is similar to that produced by the mediators of immediate hypersensitivity response (e.g., histamine, serotonin, and bradykinin), but early studies have not supported the involvement of vasoactive amines in IL-2-induced SCLS [7]. IL-2 has no direct toxic effect on endothelium but may mediate damage to endothelial cells via activation of immune effector cells. IL-2 therapy induces the production of lymphokine-activated killer (LAK) cells, interferon-*γ* (IFN-*γ*), tumor necrosis factor-alpha (TNF-*α*), and nitric oxide (NO). Natural killer (NK) and LAK cells responding to either IL-2 or IL-2-induced cytokines damage endothelial cells. While the role of IFN-*γ* in the induction of SCLS is unknown, IFN-*γ* appears in the blood of patients within 6 hours of administration of IL-2 [6, 8]. Perforin and FasL, both upregulated by IL-2, are involved in LAK cell-mediated cytotoxicity and participate in the induction of SCLS [7].

Increased levels of NO metabolites have been observed following IL-2 therapy, and IL-2 therapy leads to the induction of iNOS protein in the vascular endothelium. High NOS activity in the lungs is associated with pulmonary structural damage leading to pulmonary edema. The use of L-NAME (an NOS inhibitor) abolishes NOS activity, reduces IL-2 induced-pulmonary edema, and restores structural integrity of the lungs, suggesting that iNOS enzymes are important in the pathogenesis of IL-2-induced SCLS [6, 8]. 

Neutrophilia is commonly observed during SCLS, and neutrophils may induce endothelial cell damage. However, histopathological studies following IL-2 administration reveal lymphocyte, not neutrophil, infiltration in the perivascular tissue [7]. Other evidence suggests that IL-2 directly stimulates neutrophils and increases their adhesion to endothelial cells; damage to endothelial cells then occurs when neutrophils generate reactive oxygen intermediates, proteases, and proinflammatory cytokines (i.e., TNF-*α*.) [8] Two patients with non-Hodgkin lymphoma who presented with SCLS had elevated TNF-*α* with all other cytokine levels being normal [9].

IL-2 indirectly upregulates expression of adhesion molecules on normal lymphocytes and vascular endothelial cells. Lymphocytes cultured in the presence of high doses of IL-2 lead to the activation of NK cells and T cells. [6, 8] Cytotoxic lymphocytes use CD44 (expressed by LAK cells) to mediate endothelial injury following IL-2 administration. In addition, IL-2-induced SCLS can be mitigated by the administration of antibodies against CD44 [10]. 

In a case of idiopathic SCLS, increased vascular permeability was associated with an increase in the percentage of peripheral blood (PB) mononuclear cells carrying the Tac antigen (which identifies the *β* chain of IL-2 receptor) [11]. The increase in Tac-positive cells was closely associated with vascular leak episodes. They were present only during these episodes, and IL-2-receptors appear to be shed from the cells at the end of the episodes. IL-1 and IL-2 were not detected during attacks, possibly because a surge in IL-1 and/or IL-2 levels precedes an attack, or they may remain cell-bound and hence undetectable [11].

Compared to healthy controls, levels of the soluble form of the IL-2 receptor (sIL-2R*α*) are increased in NHL patients and correlate with disease activity. Increased levels of sIL-2R*α* were associated with the worse prognosis, presence of B symptoms, bone marrow involvement, and poor response to therapy. Levels of sIL-2R*α* decreased with disease remission, increased with disease progression or relapse, and remained elevated during nonresponse to treatment [12].

Vascular endothelial growth factor (VEGF) may also be associated with SCLS. Two patients with a chronic form of SCLS were found to have increased levels of plasma VEGF at the time of initial presentation of symptoms, and one of those patients had a decrease in plasma concentrations of VEGF with the remission of the SCLS symptoms [13]. Druey and Greipp describe, from unpublished data, finding high baseline plasma VEGF in several of their SCLS patients [14]. The VEGF protein has a known role in microvascular permeability, and elevated levels have been found in patients in septic shock [15]. The source of VEGF production and its function in the pathogenesis of SCLS remains unknown.

While most cases of SCLS described in lymphoma patients occurred as a result of anti-neoplastic therapy, this case occurred as initial presentation, resolved with antineoplastic treatment, and never recurred. To the best of our knowledge, this case represents one of only two cases of ALCL reported that presented with SCLS [16]. Unlike most cases of SCLS, our patient did not have an IgG gammopathy. [2, 4, 17] SCLS, a diagnosis of exclusion, should be considered in patients presenting with hypotension, hemoconcentration, and hypoalbuminemia; once more common causes (e.g., cardiac or liver failure, nephrotic syndrome, and sepsis) are ruled out. Identifying occult malignancy is critical, as treating the underlying malignancy may be the definitive therapy for SCLS.
